# Use of a forecast SROI analysis to evaluate a health bus intervention to help people at risk of substance use harms in Northeast England

**DOI:** 10.3389/fpubh.2026.1959188

**Published:** 2026-10-20

**Authors:** Sedighe Hosseini Jebeli, Luke Vale, Emma-Joy Holland, Amy O'Donnell, Elizabeth Titchener, Katherine Jackson

**Affiliations:** 1 Department of Applied Health Sciences, University of Birmingham, Birmingham, United Kingdom; 2 Global Health Economics Centre and Department of Health Services Research and Policy, London School of Hygiene and Tropical Medicine, London, United Kingdom; 3 Population Health Sciences Institute, Newcastle University, Newcastle upon Tyne, United Kingdom

**Keywords:** alcohol and substance use, community-based care, economic evaluation, return on investment, value for money

## Abstract

**Introduction:**

A local authority public health team in the Northeast of England worked in partnership with a group of primary care practices and a lived experience recovery organisation to deliver a Health Bus intervention to address unmet health needs of the alcohol and substance-using population. The economic evaluation of community-based interventions can be difficult to measure as there is a very broad range of impacts which goes beyond the healthcare sector. In this study, we provide an example of how Social Return on Investment (SROI) analysis enabled us to estimate the monetary value of selected outcomes through healthcare costs potentially averted.

**Methods:**

We used the forecast SROI approach. A logic model was developed by consulting local stakeholders including NGOs, charity providers and people with lived experiences. The primary outcome of interest was the referrals made through the Health Bus for the most common conditions among the target population. The time horizon was 1 year, and the perspective of the analysis was the NHS system and programme beneficiaries. The SROI was calculated based on the cost averted because of the early referrals. Additional sensitivity and scenario analysis were conducted around the parameters such as deadweight and attribution rates, productivity gain, population coverage and opportunity costs.

**Findings:**

Under the base-case with 164 participants, the estimated SROI ratio was 1.10:1, equivalent to an estimated £1.10 of social value per £1 invested. In the 4% population-coverage scenario, the estimated SROI ratio increased to 2.02:1. Results were most sensitive to changes in deadweight and attribution as well as referral rates. Relying on secondary data sources was challenging as we couldn't obtain the parameters that we needed for broader analysis, and we made certain assumptions by relying on literature.

**Conclusion:**

The findings indicate that estimated SROI ratio was highly sensitive to population coverage and attribution assumptions. The reliance on secondary data sources and assumptions, limited the analytical scope focused only on unmet health needs. Future studies can focus on broadening the analysis to incorporate non-health outcomes by primary data collection and tailored surveys in a longer implementation period, which then allows for projections beyond the pilot duration.

## Background

Across the United Kingdom (UK), people who use alcohol and other substances have well-documented unmet physical and mental health needs (1–3). They often experience significant unmet health needs, with many not engaged in treatment despite high levels of morbidity and social disadvantage (3). These gaps are particularly evident among socially excluded groups, such as people experiencing homelessness, who may face additional barriers to accessing services such as a stable home address for correspondence and the financial resources for travel to appointments (1). Individuals with co-occurring mental health and substance use conditions are further disadvantaged by the challenges they face in navigating fragmented care systems and conflicting eligibility criteria, which can lead to exclusion from appropriate support (4). Overall, this population commonly presents with complex, overlapping health and social needs which highlights the importance of accessible, community-based interventions to better address their needs (5).

In one local authority area based in Northeast England, a programme was delivered to address the unmet health needs of this population with embedded research and evaluation (6). A co-production model, informed by co-design principles, was adopted to enhance acceptability, trust, and relevance among the target population. Embedding a mixed-method evaluation at an early stage enabled systematic mapping of both health and non-health outcomes and the subsequent monetary evaluation through Social Return on Investment (SROI) analysis. The SROI component estimated the monetary value of outcomes associated with addressing unmet health needs and compared these to the costs of delivering the intervention.

In an era of increasing healthcare demand and constrained public finances, funders and commissioners require robust evidence of value for money (VfM). Traditional economic evaluation approaches, including cost-effectiveness analysis and cost-utility analysis have long been used to inform resource allocation decisions. However, SROI has emerged as an approach that seeks to capture a broader spectrum of value, incorporating health and non-health outcomes across social and economic domains (7). By identifying, measuring, and assigning monetary values to outcomes that matter to stakeholders, SROI aims to provide a more comprehensive account of social impact.

SROI is grounded in a framework that values change from the perspective of those who experience it. It also considers how much of those changes would have happened anyway (deadweight) and how much can be credited to the programme (attribution) (7). This study obtained deadweight and attribution estimates through a specified elicitation process. It has been applied across public health and community interventions to demonstrate the societal value of services beyond direct healthcare savings (8). In the context of alcohol and substance use, where impacts extend to employment, housing stability, and service utilisation across multiple sectors, such a holistic evaluative approach is particularly useful.

There are two main forms of SROI: evaluative SROI, conducted retrospectively using observed outcomes, and forecast SROI, which estimates expected social value based on measured and projected outcomes (7). This study aimed to conduct a forecast SROI analysis informed by observed pilot service outputs and modelled downstream outcomes over a 1-year time horizon. This enabled an estimation of the anticipated longer-term social value generated by the intervention.

### Pilot study design

By acknowledging the urgent need to reduce health inequalities among People Who Use Substances (PWUS), the Mobile Health Clinic which from here will be referred to as the Health Bus, was established to improve access to primary healthcare for individuals at heightened risk of alcohol- and substance-related harm. PWUS frequently experience substantial unmet physical and mental health needs, coupled with barriers to mainstream primary care, including stigma, unstable housing, and fragmented service engagement. The Health Bus was therefore designed as an outreach-based model to bring essential healthcare directly to community settings familiar to the target population.

The service delivered core elements of primary care, including blood pressure monitoring, assessment and management of minor acute conditions such as wounds and infections, and provision of health information, advice, and onward referral to specialist services where appropriate. The intervention was accessible to individuals at risk of harm related to alcohol or substance use, including those experiencing homelessness or social exclusion, as well as others present at the community locations where the bus was stationed.

The bus was operated by staff employed by a group of primary care practices and a lived experience recovery organisation and was delivered through a multidisciplinary team model. Each session included a clinician (either a GP or Advanced Nurse Practitioner), an administrator responsible for registration and triage, and a service manager overseeing operational delivery, all from primary care. Importantly, the service incorporated relational peer workers with lived experience from the lived experience recovery organisation. Peer workers played a central role in engagement and trust-building, supporting individuals to access the bus, facilitating registration, and linking attendees to wider health, social care, and community support services beyond immediate clinical needs. This integrated and person-centred approach aimed not only to address acute health concerns but also to strengthen pathways into secondary care, drug and alcohol treatment, community and voluntary sector provision (e.g. food banks), housing and benefits support. However, all referrals were made to the secondary care to address the immediate health needs and the SROI analysis also focused on this aspect to measure the downstream benefits.

## Methodology: SROI approach

### Scope of the study

#### Population

The core evaluation cohort for the primary analysis was the number of patients who self-referred to the bus and received care from the multidisciplinary team. However, to explore the potential impact of future scale-up, the broader local population in need was also estimated to model alternative implementation scenarios. To estimate the size of the population in need, percentage of adults with high-risk drinking (9), was applied to the adult population of the relevant local authority area. High-risk drinking was selected because it captures a broader segment of the population at-risk compared with other criteria such as illicit drug use or homelessness alone. This approach therefore provides a more inclusive estimate of the potentially eligible population and enables modelling of scale up scenarios reflecting wider unmet need across the local authority area.

#### Intervention and outcomes

The bus delivered core elements of primary healthcare directly to the PWUS population as described earlier. The primary objective of the intervention was to address unmet health needs among the PWUS population by providing immediate, accessible stigma-free primary healthcare and facilitating onward referral where appropriate. Accordingly, outcomes included observed health service contacts and referrals generated through the intervention. However, for modelling purposes some the below outputs and outcomes were projected as well.

1. Observed inputs and outputs: Costs, attendees, consultations, treatments and referrals.
2. Reported immediate outcomes: Satisfaction and access.
3. Projected outcomes: Referral uptake, treatment effects and avoided hospital use.

#### Logic model

Following stakeholder consultations through regular Community Advisory Board (CAB) meetings (e.g. with community service providers and people with lived experience), and consistent with the scope of service delivery, a logic model (Figure 1) was developed to describe the anticipated pathways through which the intervention could generate impact. This model captures almost all the possible health and social outcomes which could have been ideally measured if data were available. However, the intervention did not directly aim to produce or measure sustained behavioural changes. Consequently, broader non-health outcomes, including changes in crime rates or long-term social functioning, were not included. Survey and semi-structured interviews were conducted with PWUS participants who accessed the bus (6). However, family members and carers were not directly surveyed or interviewed. As a result, their perspectives and associated social value were not incorporated into the SROI estimates (see below).

**Figure 1:**
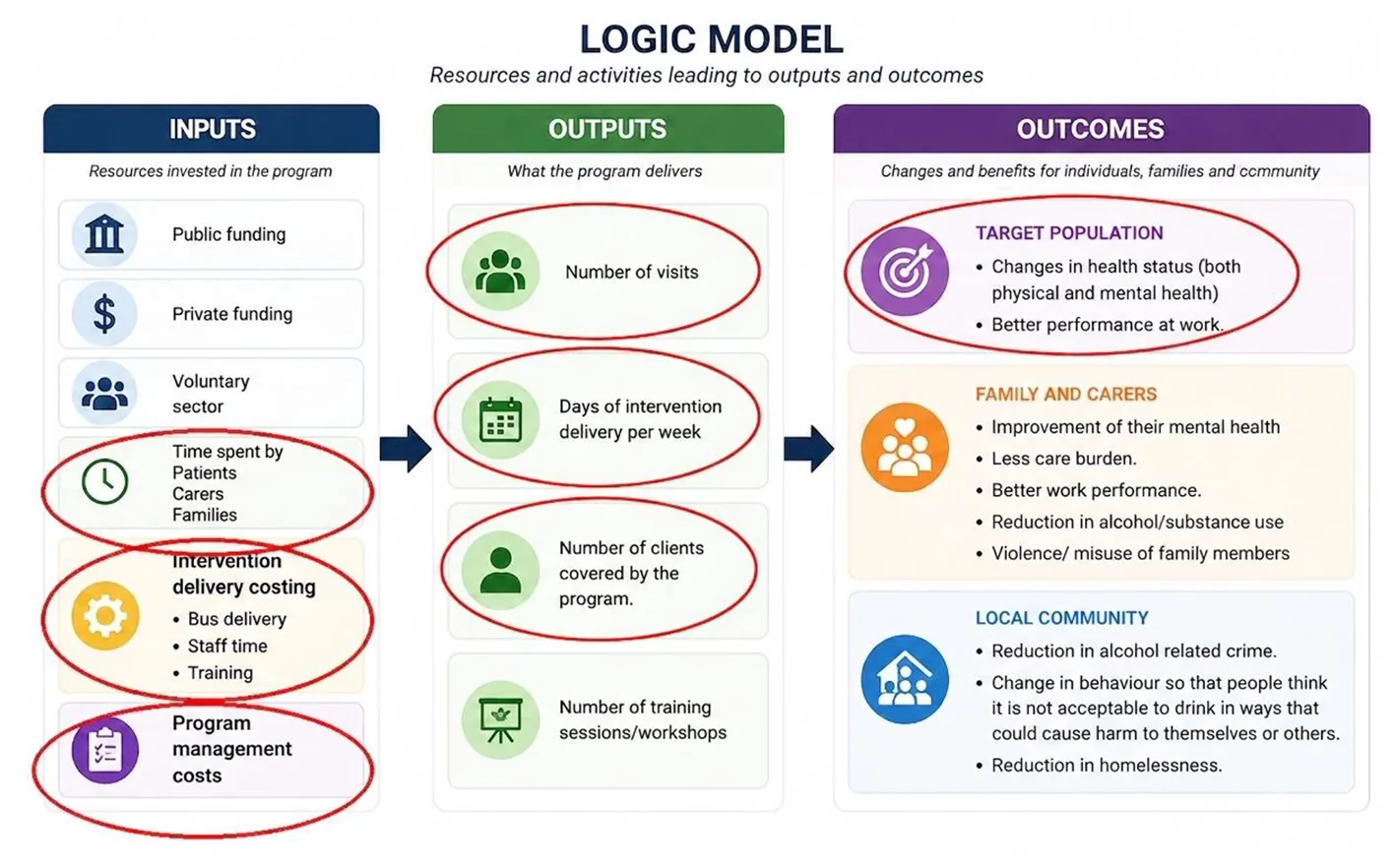
The logic model: the ones circled are measured and monetised in the SROI model.

#### Setting

The intervention was delivered from 1 April to 31 October 2025. The bus was operating two days per week with a month break during August. During this time, the Health Bus operated across a range of community-based locations in a city in Northeast England, including church-based hubs offering drop-in kitchens for individuals experiencing homelessness or food insecurity, food banks, community centres, and voluntary sector recovery organisations. This outreach model was designed to maximise engagement with individuals who may be underserved by conventional primary care provision.

### Data collection

For the purposes of the SROI analysis, the primary quantitative sources were a patient survey, administered to patients by the research team immediately after patients attended their consultation on the health bus and routine patient data collected by the GP providers, and then anonymised and shared with the research team (6). These datasets were complementary, with partial overlap as not all patients who accessed the bus completed the survey (see the findings for details). It should be noted that survey data were not linked to the routine data as the survey was filled anonymously and for confidentiality reasons, we couldn't track the individuals in routine data to ensure the completeness. We obtained healthcare utilisation patterns and user experience from survey while routine GP data provided us with substance use history and experiences of homelessness, clinical information such as health conditions, treatments delivered, and referrals generated through the Bus. Together, these data sources enabled quantification of existing conditions and referrals.

### SROI projection and analytical perspective

A *de novo* Excel model was developed and outcomes were projected over a 1-year time horizon following implementation of the intervention. We followed all six stages of SROI (7) from scoping, mapping the outcomes and evidencing the outcomes to establishing the impact and calculating the SROI ratio. However, as elaborated earlier, we had limited access to data, and we only monetised the referrals made and downstream productivity benefits.

Total costs and benefits were estimated by modelling the expected annual number of PWUS individuals accessing the service and receiving early assessment, treatment, or referral for five key health conditions: COPD, Asthma, Anxiety and depression, musculoskeletal pains; identified through survey and clinical data. It should be noted that selected downstream benefits (e.g. avoided hospitalisation) were projected from observed referrals using secondary evidence and assumptions.

The SROI analysis focused on the immediate healthcare gaps and was conducted from the perspective of patients and the National Health Service (NHS) in terms of productivity and hospitalisation costs averted respectively. Impacts on carers, families, and wider non-health sectors, such as housing or criminal justice were therefore not included in the valuation. While this approach ensures alignment with service objectives, it could result in an underestimation of the broader societal value associated with improved healthcare access among this population.

### Resources and cost estimation

Resource inputs for both the pre-implementation and operational phases were identified and costed using programme budget reports through a micro costing approach. Pre-implementation costs included planning, training, and management activities required to establish the service. Operational costs comprised staffing, administrative support, and delivery expenses associated with running the bus. This includes hiring the bus with all associated cost (insurance, fuel etc) and in the scale up scenario we increased the operations days to 4 days a week per bus. It should be noted that we didn't account for the peer-worker time present in the bus.

Estimates of cost savings (costs averted per person using the bus) were derived from published sources (10–13) which reported the burden of disease and cost per each patient/hospital stay. The values were then adjusted for inflation to the 2025 price year reported in British pound. These cost savings primarily reflected avoided secondary care utilisation resulting from early identification and treatment of health conditions as shown in the pathway below:

observed referral → assumed reduction in hospitalisation → estimated cost averted.

### Outcome identification and evidencing

Primary outputs were the number of individuals treated on the bus and the number of referrals made to other health services (Figure 2). The main pre-existing conditions managed through the service were identified through descriptive analysis of survey responses and routine data from the GP practice partner.

**Figure 2:**
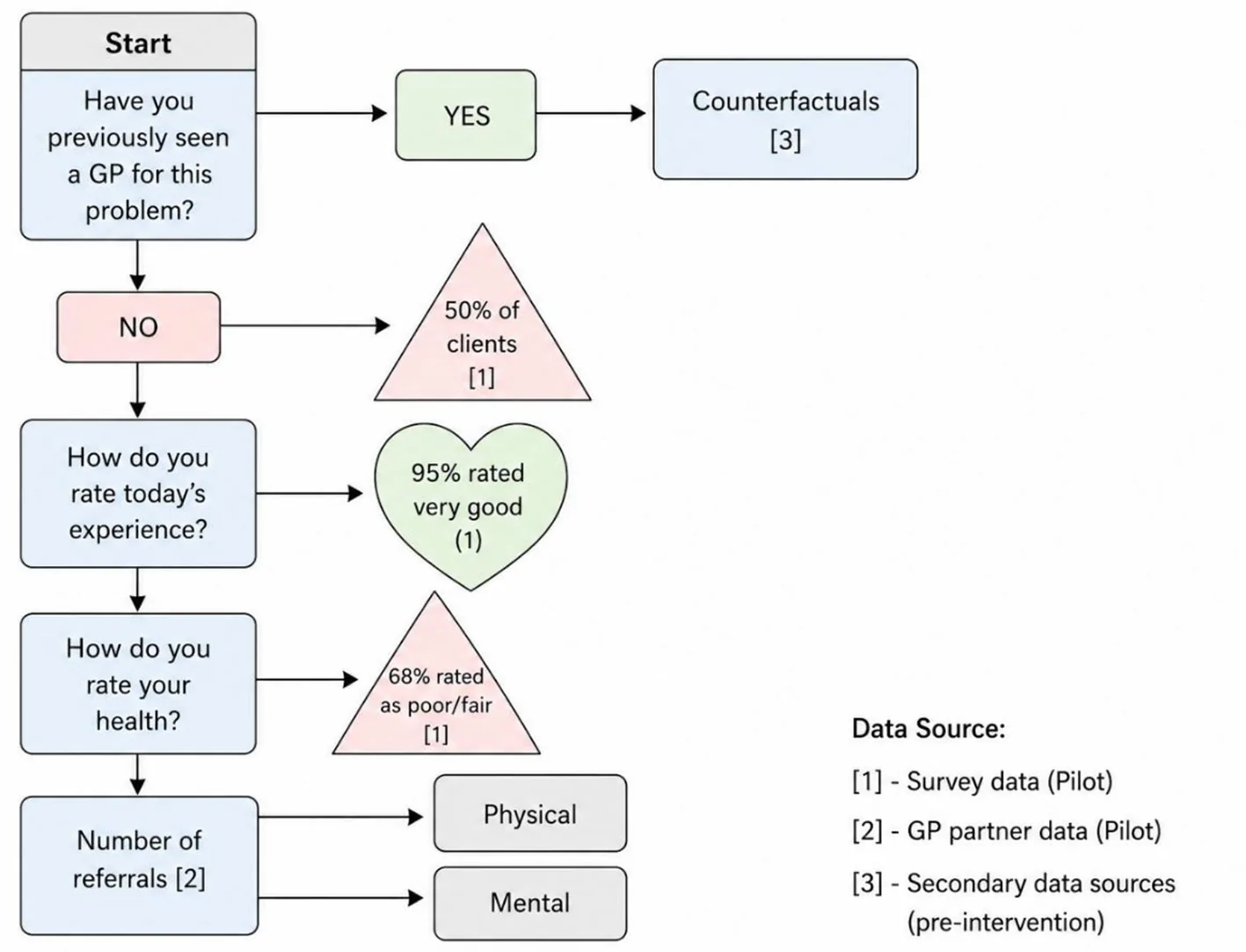
Quantifying service outputs (all values are observed and measured during the pilot phase).

Secondary outcomes included participant-reported satisfaction and improved access to General Practitioner (GP) services. Improved access was derived from the survey by asking if the clients have been to GP before because of their existing condition. Productivity gains associated with improved health status and reduced hospitalisation were incorporated within the scenario analysis as described above, rather than the main (base-case) model.

### Outcome valuation

The main financial proxy applied to referrals and primary care delivered on the bus was the estimated cost averted per person using the bus. It should be noted that the condition-specific financial proxy was applied to referrals.

Avoided costs were calculated by estimating the probability of hospitalisation for relevant conditions and multiplying this by the average annual cost of hospital admission per patient. This approach estimates potential reductions in secondary care utilisation under assumptions about referral uptake, treatment and subsequent hospitalisation risk (see above).

Productivity gains were quantified using national statutory sick leave pay as a proxy and estimated reductions in hospital stays and sickness absence among the target population. We used national statutory sick leave pay rather than the average wage because the target population face more barriers to enter the labour market, experience intermittent employment, or are employed in lower-paid or part-time work. Applying the average national wage would therefore be likely to overestimate the economic value of productivity gains for this population. Statutory sick leave pay provides a conservative estimate of the minimum economic value associated with avoided sickness absence and reflects a more realistic proxy for the income replacement available to many individuals in this group. Consistent with survey findings, an employment rate of 6% was applied to the cohort when calculating productivity-related benefits.

### Impact calculation

To improve the robustness of the SROI estimates, key impact-adjustment factors were incorporated into the analysis. These included:

- **Deadweight**, representing the proportion of outcomes that would have occurred without the intervention
- **Attribution**, representing the proportion of outcomes attributable to other services or programmes
- **Duration**, reflecting the expected persistence of outcomes over time

Estimates for deadweight and attribution were informed through structured consultation with stakeholders participating in the Community Advisory Board (CAB) held online on November 2025 with 20 people in attendance including the Local Authority team, community service providers and people with lived experiences. Stakeholders were presented a brief summary of SROI analysis and impact measures in lay language with specific question followed: “what could have happened without the intervention?” Then they were asked to propose plausible rates for deadweight and attribution based on their professional and lived experience. All the rates were recorded, and we used the average rate for both deadweight and attribution in our base case. However, sensitivity analyses were conducted using alternative deadweight and attribution values (upper and lower ranges) to test the sensitivity of the overall SROI ratio to variations in these parameters.

### SROI ratio calculation

Social Return on Investment (SROI) was calculated using the following formula:


$$\begin{array}{l} \text{SROI}=\text{SIV}/\text{IIA while the SROI net return percentage was}\\ \;\text{calculated via SROI }=\left[\left(\text{SIV}-\text{IIA}\right)/\text{IIA}\right]\;\times \;100 \end{array}$$

Where:

- **Social Impact Value (SIV):** the aggregate monetised value of outcomes generated by the intervention within the defined time horizon
- **Initial Investment Amount (IIA):** the total financial resources required to design, implement, and operate the bus

The resulting ratio represents the net social value estimated relative to the initial investment. A value greater than one indicates that the social value created exceeds the cost of delivery.

### Sensitivity analysis

A one-way sensitivity analysis was conducted to assess the robustness of the base-case results. Key parameters were varied independently using different ranges while holding others constant. The analysis demonstrated even relatively narrow variation in cost and outcome materially affects the SROI ratio. The following parameters and ranges are reported:

1. **Outcome magnitude:** changed by ±5%
2. **Deadweight and attribution:** varied by ±10%−15%
3. **Cost per patient per year:** adjusted by ±5%
4. **Referrals rate: adjusted by** ±5%

### Scenario analysis

Three scenario analyses were undertaken to explore broader system implications beyond the base-case model:

- **Population coverage:** modelling increased the reach across the eligible population in Sunderland.
- **Opportunity cost:** estimating the value of alternative resource use which was then subtracted from the estimated benefits in a separate scenario.
- **Productivity gain:** estimating economic benefits associated with avoided hospital stays and reduced sickness absence.

#### Population coverage

To generate the scale up scenarios, we needed to estimate the population in need of this intervention. As the exact PWUS population in Sunderland is not available in existing reports, we multiplied the high-risk drinking percentage (9) by total Sunderland population based on 2021 census data (adjusted in early 2026) to estimate population in need. The pilot intervention covered 0.04% of this population and we used 2% and 4% as scale up scenarios.

#### Productivity gain

For scenario modelling, COPD was considered an important contributor to hospitalisation among people who use substances because respiratory disease is substantially more common in this population, and individuals with COPD experience significantly higher rates of COPD-related hospital admissions than the general population (14). National data indicate that patients aged under 65 accounted for approximately 23% of COPD admissions in England in 2024, with an average hospital length of stay of 5.03 days (15). A 2025 analysis estimated the average cost per COPD hospital bed day at £261 (16).

The total hospitalisation cost avoided was calculated as:


$$\begin{array}{l} \text{Total hospitalisation cost avoided }=\text{ Average length of stay}\\ \;\times \text{ Average daily hospital cost}\\ \;\times \text{ hospital admission rate}\\ \;\times \text{ target population} \end{array}$$

To estimate productivity gains associated with reduced sickness absence:


$$\begin{array}{l} \text{Total sick leave cost avoided }=\text{ Average days of sick leave}\\ \;\times \text{Cost per sick leave day}\\ \;\times \text{ employment rate}\\ \;\times \text{ target population} \end{array}$$

Evidence indicates that employed individuals with mild-to-moderate COPD experience significantly higher sickness absence than the general UK workforce, averaging 16.3 days per year (17). The value of each sick leave day was derived from the UK Statutory Sick Pay rate of £118.75 per week as of April 2025, converted to a daily equivalent (18).

An employment rate of 6% was applied to the PWUS population, consistent with survey findings. The total productivity gain across different sectors was calculated by summing avoided hospitalisation costs (from NHS perspective) and avoided sick leave costs (Department of Work and Pension). This approach provides a conservative estimate of productivity-related benefits given the low employment participation and short analytical time horizon.

### Opportunity cost

In the absence of the bus, GP consultations were considered the primary alternative pathway through which PWUS population might have their health needs addressed, as based on our survey 50% of them have been to GP before for their existing condition. This reflects the possibility that half of the cohort might otherwise have accessed care through routine primary care pathways

The opportunity cost was therefore defined as the estimated cost of GP consultations that could have otherwise provided assessment and referral. Accordingly, the opportunity cost was calculated by multiplying the unit cost per GP consultation by 50% of the study population and then subtracted from the estimated total benefits in a separate scenario.

## Result

Under the base-case modelling assumptions, the intervention was estimated to generate a positive social return over the 1-year analytical horizon. The estimated base-case SROI ratio was 1.10:1, corresponding to £1.10 of estimated social value per £1 invested, or a 10% net return.

The Figure 3 shows the number of total clients self-referred to bus, survey respondents and those referred for further care. The population characteristics, common health conditions and impact calculations are presented in Tables 1–3 respectively. SROI findings are reported below in Table 4 for the base-case analysis, followed by sensitivity (Table 5) and scenario analyses (Table 6). These latter two sets of analyses explore sensitivity to alternative assumptions. Outcomes are presented in terms of quantified service outputs, monetised benefits, and the resulting SROI ratio, which provides an overview of the value for money generated by the intervention within the 1-year analytical time horizon.

**Figure 3:**
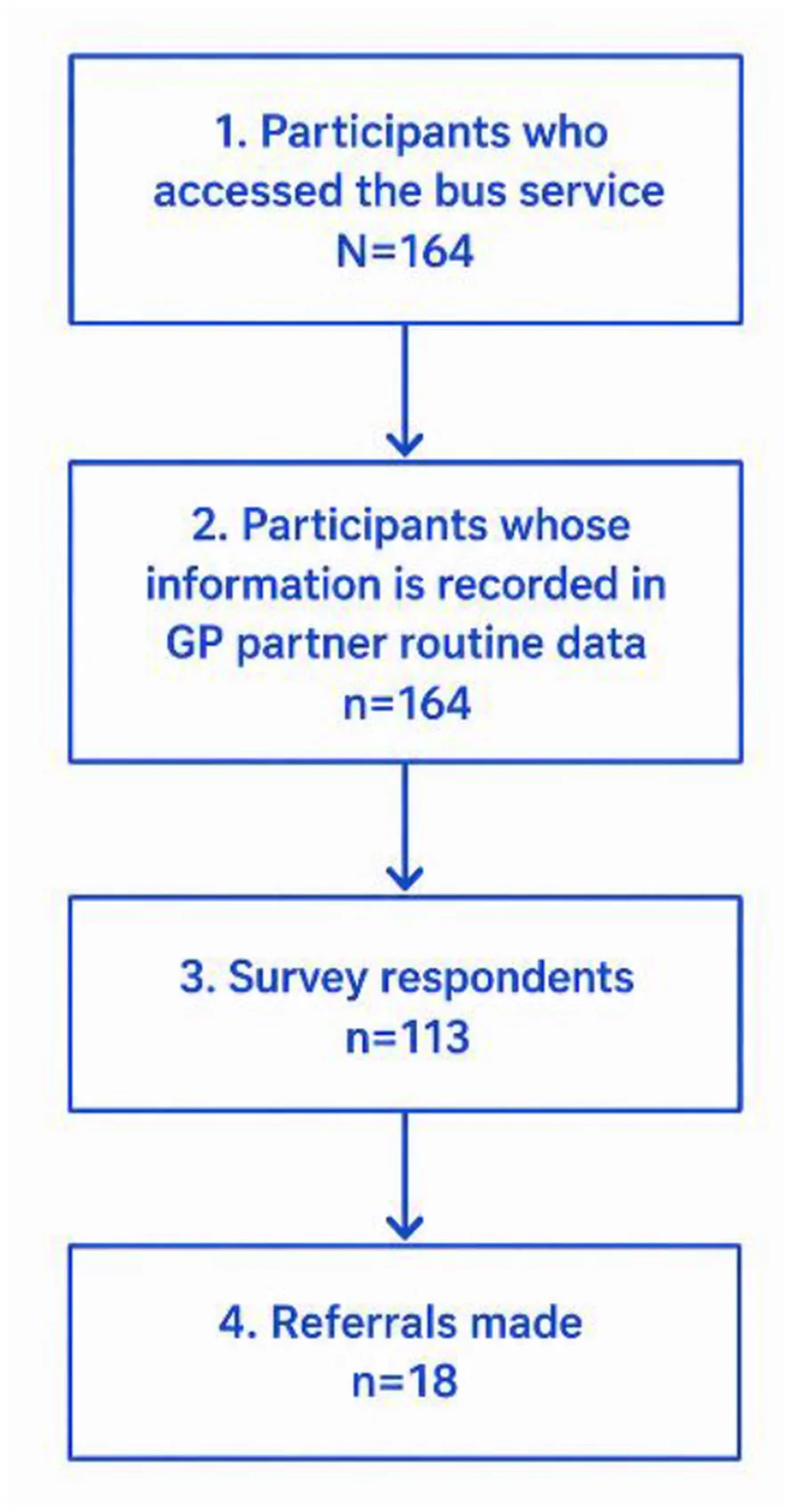
The data flow chart.

**Table 1:** Sociodemographic characteristics of the pilot population.

Parameter	Frequency	Percentage	Range	Average
Total participants	164	NA	NA	NA
Gender				
Male	115 (164)	70%	NA	NA
Female	49 (164)	30%	NA	NA
Age	NA	NA	17–92 years	48
Ethnicity				
White/mixed British	148 (164)	90%		
Missing data	16 (164)	10%		
PWUS population				
History of alcohol misuse	65 (164)	40%		
Experiencing homelessness	55 (164)	34%		
Homeowner/rental	96 (164)	59%		
Deprivation index				
IMD 1 and 2^*^	62 (96)	65%		

Source: GP partner routine data collected at the point of service.

^*^The denominator for IMD is 96 as 96 out of 164 clients were either homeowner or renting a place with permanent address.

**Table 2:** Health status, common conditions and referrals.

Parameter	Frequency	Percentage
Diagnosed mental or physical condition^*^	99 (113^**^)	88%
Asthma and COPD	15 (113)	13%
Depression/anxiety	57 (113)	50%
Blood pressure and diabetes	12 (113)	11%
Rating of bus experience		
Good	26 (113)	23%
Very good	82 (113)	73%
Referral made^***^	18 (164)	11%

Source: Survey and GP routine data.

^*^Some clients had co-existing conditions. Each of the conditions were counted separately.

^**^Denominator for survey is 113 respondents.

^***^Referral rates coming from GP routine data.

**Table 3:** Impact calculation.

Indicator	Observed referrals/prevalence rate	Probability of averted hospitalisation (assumed)	Changes per person (estimated)	Deadweight	Attribution
COPD referrals^*^	11:00%	36:00% (19)	1.98%	25:00%	25:00%
Asthma Referrals^*^	11:00%	32.10% (20)	1.76%	25:00%	25:00%
Anxiety and depression^*^	11:00%	19:00% (21)	1.04%	25:00%	25:00%
Musculoskeletal pains^**^	31:00%	44:00% (22)	13.64%	25:00%	25:00%

^*^Changes per person is calculated based on the percentage of overall referrals made (observed) and the averted hospitalisation rate for each condition (assumed) per this formula: referrals rate^*^expected average reduction in hospitalisation^*^50% (counterfactuals).

^**^Changes per person for musculoskeletal pain = observed prevalence^*^probability of reduction in hospital cost.

**Table 4:** SROI ratios—base case scenario (pilot intervention).

Indicator	^**^Value generated (estimated) £	Cost averted per person (per literature) £	Total investment (reported by actual finances) £	SROI ratio
Increased number of COPD referrals	74,835.58	40,971.00 (12)^*^	102,473.00^***^	1.10
Increased number of Asthma referrals	29,858.48	18,333.00 (13)		
Increased number of depression and anxiety referrals	5,494.87	5,700.00 (11)^*^		
Musculoskeletal pains treated at the point of service	2,527.28	200.85 (10)^*^		
Total	112,716.20	—		

^*^The cost per person are informed by the literature (per citations).

^**^The value generated considers the cost averted in the population and is modelled and estimated.

^***^Total investment is the amount that was spent on the intervention reported by the local authority.

**Table 5:** One-way sensitivity analysis.

Each parameter	Lower range	SROI ratio	Upper range	SROI ratio
Cost averted per person	−5%	1.04	+5%	1.15
Changes in Outcomes	−5%	0.94	+5%	1.26
Deadweight	15%	1.25	40%	0.88
Attribution rate	15%	1.25	40%	0.88
Referral rate	−5%	0.61	+5%	1.59

The referral rate has the biggest impact on the SROI ratio (ranging from 0.61 to 1.59 with a 5% shift), while changes in outcomes also cause high volatility around the break-even point. Deadweight and attribution share the same sensitivity profile, dropping the SROI to an unhealthy 0.88 if they rise to 40%. Cost averted per person is the most stable parameter, keeping the SROI above break-even (1.04) even with a 5% decrease.

**Table 6:** Scenario analysis.

Each scenario	Population covered	Total SROI ratio
1. Population coverage	In need: 37,584^*^	
4%	1,503	2.02
2%	752	1.68
2. Opportunity cost	NA	1.07
3. Productivity gain	NA	1.07

^*^Population in need is estimated (see above: population coverage estimates).

In modelling, the inputs required and associated costs were also adjusted proportional to population coverage.

The referral rate as a key input remained constant similar to observed rate in pilot phase.

The scenario analysis, shown below in Table 6, demonstrates that increasing population coverage (see above: population coverage) could improve value generation, for example operating the bus on additional days.

## Discussion

This study provides one of the first SROI analyses of a multidisciplinary mobile health clinic for PWUS to our knowledge. Guided by a logic model, the Social Return on Investment (SROI) analysis estimated a base-case ratio of **1.10:1**, indicating that every £1 invested generated £1.10 in return. The logic model provided a structured framework for identifying how resources invested in the intervention, such as staff time, intervention delivery, and programme management costs, translated into service outputs (e.g., number of visits, intervention days, and clients reached) and projected health and social outcomes. However, due to data availability and the limited evaluation timeframe, it was not possible to measure or monetise all elements of the logic model. Consequently, the SROI analysis focused on those inputs, outputs, and outcomes for which the data were available, and the estimated social value is likely to represent a conservative assessment of the intervention's overall impact. In fact, excluded benefits may increase estimated value, but omitted costs, failed referral uptake or weaker treatment effects may decrease it.

Although the base-case model estimated a positive net return, sensitivity analyses produced SROI ratios below 1.0 under plausible alternative assumptions.

Such conservative returns are typical of early-stage community and primary healthcare initiatives, where limited scale and short evaluation periods often constrain measurable value (7, 8). Expanding the analysis to include the broader outcomes identified within the logic model, such as improved wellbeing, increased employment, enhanced social participation, reduced caregiver burden, and reductions in criminal justice involvement, could have captured additional sources of value generated by the intervention. Incorporating these broader social and economic benefits would likely have increased the estimated SROI, as a wider range of positive impacts beyond direct health gains would be monetised and reflected in the overall return. However, additional operational costs, failed referrals or treatment uptake would have decreased the estimated return on investment values.

Projected social value in this analysis was driven by assumptions attached to the increased number of referrals for chronic respiratory conditions, particularly chronic obstructive pulmonary disease (COPD) and asthma. Within the logic model, these referrals represent key program outputs that contribute to the projected downstream health outcomes and subsequent economic value. Early identification and timely referral for long-term respiratory conditions are well-established as cost-saving strategies, primarily through reductions in emergency admissions and exacerbations and this fact has informed our modelling and analysis (23, 24). Evidence from integrated respiratory care models within the National Health Service has demonstrated that proactive case finding and structured management pathways reduce unplanned hospital utilisation and improve disease control (25) and this was one of the aims of the pilot intervention.

Mental health referrals for depression and anxiety generated comparatively lower monetised value in our modelled estimation. However, extensive evidence demonstrates that early access to psychological therapies leads to significant long-term health and economic benefits, including improved employment outcomes and reduced healthcare utilisation (26, 27). These wider benefits align with the longer-term outcomes described in the logic model but were only partially captured in the current SROI. National evaluations of the Improving Access to Psychological Therapies programme have shown recovery rates exceeding 50%, alongside measurable economic gains through reduced welfare dependency and increased productivity (28).

Sensitivity analysis showed that the SROI ratio was most affected by variations in outcome magnitude, deadweight, and attribution. For instance, reducing the estimated outcomes by 5% lowered the SROI ratio to 0.94. Additionally, the analysis highlights how sensitive the findings are to assumptions surrounding deadweight and attribution, which capture the counterfactual of what would have occurred without the intervention (7, 29).

Scenario analysis demonstrated that increasing population coverage substantially improved value generation. Expanding reach from pilot level to 4% of the eligible population increased the SROI ratio to 2.02, which could be a reflection of economics of scale observed in integrated care initiatives (30, 31). This suggests that high up-front costs could be offset if the bus operates more days per week and reaches a larger proportion of the eligible population although productivity gains were exploratory projections, they are similarly supported by evidence linking improved health outcomes to increased workforce participation and reduced absenteeism (27).

## Limitations

This study provides an initial estimate of the social value generated by the intervention using a SROI approach. However, several important limitations should be considered to replicate the work in future.

- **Lack of Referral Follow-Up:** Participants were not followed beyond their initial health bus visit, making it impossible to determine whether referrals resulted in service attendance or subsequent health improvements. Consequently, the analysis relied on assumptions regarding referral uptake, introducing uncertainty into the estimated values.
- **Data and Outcome Gaps:** Several longer-term outcomes, including sustained recovery, improved wellbeing, housing stability, and reduced criminal justice involvement, could not be monetised due to limited data availability.
- **Uncertain Scale-Up Costs:** The financial modelling does not fully account for the variable and overhead costs associated with scaling the intervention beyond its current pilot capacity.
- **Population Mismatch:** Since the exact PWUS population estimates are not available, high-risk drinking population in our scale up scenarios as a proxy for PWUS which can over/underestimate the used in associated impacts.

Future studies should incorporate longer-term follow-up (e.g. 12 months or beyond) and cross-sector data linkage to provide a more comprehensive and robust estimate of social impact.

## Conclusion

Under the base-case modelling assumptions, the programme was estimated to generate a positive social return over 1 year. The modest base-case ratio reflects the selected base-case assumptions, short follow-up duration, and the exclusion of broader indirect benefits such as improved wellbeing or reduced criminal justice involvement. Future work should distinguish modelled cost offsets from cash-releasing savings and identify which organisations bear the intervention costs and which receive the benefits.

Although longer term, comprehensive evaluation is needed, we believe that extending the bus operation days per week could compensate for the high up-front costs and allow more people to access the service, which would result in a higher return on investment. Work should also identify achievable savings as distinct to free up capacity for services. It should also identify clearly who gains the benefits/savings and who bears the costs.

## Data Availability

The datasets presented in this article are not readily available because of the small and identified study area which would make participants potentially identifiable. Requests to access to additional summary data should be directed to the corresponding author.

## Glossary

Attribution: The extent to which observed outcomes can be directly linked to the intervention, rather than to other external factors or influences.

Deadweight: The proportion of outcomes that would have occurred anyway without the intervention.

Duration: The length of time over which the outcomes of an intervention are expected to last.

Perspective: The viewpoint adopted in an economic evaluation that determines which costs and outcomes are included (e.g., healthcare system, societal, or patient perspective).

PWUS population: Our target population for this study. This term refers to people experiencing drug and alcohol related harms, who are in recovery from drug and alcohol related harms, and/or who experience homelessness (past or current).

Productivity: The level of output or economic contribution produced by an individual, often measured in terms of work capacity, employment, or efficiency.

SROI (Social Return on Investment): A framework for measuring and valuing the broader social, economic, and environmental outcomes generated by an intervention relative to the investment made.
